# Intemperance in Europe and the East

**Published:** 1857-04

**Authors:** J. Oscar Noyes


*Intemperance in Europe and the East.* By Dr. J. Oscar Noyes.

There is nothing truer under the sun than the old adage, *de gustibus non disputandum.* When I was a votary of the scalpel under Hyrtl, of Vienna, that great naturalist used to bemoan the loss of an immense collection of anatomical specimens, worth several thousand dollars, at the capture of the city by the Imperialists in 1849. The Croats drank off the alcohol in which the preparations had been preserved, and trampled the professor's household gods under their feet. The anatomical cabinet of Professor Ruysch and daughter, of Amsterdam, was so admired by Peter the Great, that he purchased it at the cost of 36,000 ducats. The preparations were shipped safely to Cronstadt, but in passing from the latter place to St. Petersburg the alcohol was spirited away, as in Vienna, and the collection totally ruined.

The Russians are in fact enormous consumers of alcohol. They send it against the Circassians as they have sent their Crabbes and Woronzoffs. The government derives a great part of its revenue from the excise levied upon intoxicating drinks. At the commencement of every term of the University of St. Petersburg, the servants (old soldiers who have distinguished themselves in the service of their country) are brought forward, and in their presence a portion of arsenic is introduced into every bottle containing an anatomical preparation. Even then they are required to take the oath of total abstinence. In Russia we might almost term alcohol "a fourth estate"—"a power behind the throne, stronger than the throne itself.

I think that travelers making a hasty tour of the continent are generally guilty of egregious errors in their estimate of the extent of intemperance in Europe, and its effect upon social institutions. They see comparatively few drunkards in the streets and, without caring to make a closer examination, are ready to affirm that intemperance scarcely has an existence—that France, Italy and Germany constitute the Utopia of modern Drinking. During a year's residence in Vienna, I had abundant opportunity to observe the extent to which alcoholic drinks were there used.

There are comparatively few drinking-houses after our model in the Austrian capital, but the *gast-hauser* serve as lager-beer saloons, and *liquors* are sold largely in the thousand and one cafés.

I am of the opinion that in Germany, a greater portion of the people indulge in alcoholic drinks, aside from wine and beer, than is the case among ourselves.

In the quarter of Vienna where I resided, one of the most genteel of the city, scarcely a Sabbath evening passed without drunken brawls, to end which, the interference of the police was often necessary.

Cases of death resulting directly or indirectly from intemperance, as reported in the journals, were of very frequent occurrence. I remember in particular, two remarkable instances of sudden death, occasioned by drinking large quantities of spirituous liquors.

The wards of the General Hospital contained about the same proportion of patients whose disease, hepatic and the like, had been induced by intemperance, as the hospitals in the United States.

Cases of delirium tremens were of frequent occurrence. One of the most common and obstinate forms of disease was distension of the stomach, caused by drinking inordinate quantities of wine and beer. So common was this, in fact, that some of the attending physicians used ordinarily to begin the examination of newly arrived patients by percussing them over the region of the stomach. I may state, *en passant*, that some of the moderate Germans boast of being able to drink a gallon of wine per day, and glasses of Teutonic ale too numerous to mention. Professor Oppolzer, a physician of world-wide celebrity, whose *cliniques* I attended, often deprecated the drinking habits of the Germans as highly deleterious to health and general happiness. And, in my opinion, the Viennese are more unhealthy and short-lived than the inhabitants of New York.

Hyrtl, by far the greatest anatomist living, often said to us, that by a single stroke of the scalpel, in the dark, he could distinguish the brain of an inebriate from that of a person who had lived soberly. Now and then he would congratulate his class upon the acquisition of a drunkard's brain, admirably fitted, from its hardness and more complete preservation, for the purpose of demonstration. As is well known, when the anatomist wishes to preserve a human brain for any length of time, he effects his object by keeping the organ in a vessel of alcohol. From a soft, pulpy substance, it then becomes comparatively hard. But the inebriate, anticipates the anatomist, while the brain remains the consecrated temple of the soul—while its delicate and gossamer tissues still throb with the pulse of heaven-born life. Terrible enchantment, that dries up all the fountains of generous feeling, petrifies all the tender humanities and sweet charities of life, leaving only a brain of lead and heart of stone!

Vienna is one of the most dissolute capitals of Europe. The low morality—the indecencies exhibited on every hand—the universal desecration of the Sabbath—half of the births in the city illegitimate—the troops of legalized prostitutes—a single lying-in hospital, to which a married female can scarcely procure admission, producing annually between four and five thousand bastards for death in infancy, or prostitution and conscription in later years. Would all these things be as they are if the Viennese were a temperate people? No one, I believe, can resist the conclusion, that in their drinking habits is the foundation of most of these evils.

Were the Germans to dispense with some of their wine, beer, and *liqueurs*, they would, in my opininion, become more clear-headed, sound-hearted, and virtuous. The despotic, anti-liberal tendency of their literature would cease to exist, and German infidelity—that muddy stream of presumption and pedantry—would, when viewed in clear light, become more repulsive and less injurious. With them the grosser appetites are suffered to

> "—— Drink up the liberal sap,
> The vegetating vigor of philosophy,
> And leave it a mere husk."

Moreover, wine and beer, with music and the concomitant pleasures, all furnished at a cheap rate, are acknowledged to be important means in the hands of German tyrants for keeping their subjects oppressed. Casks of lager-beer, butts of wine, and fiddle bows—the props of despotism and the upholders of dynasties! This is not saying much for the gallantry and heroism of the people; nor, in my opinion, are they worthy of liberty who are willing to take in exchange for it the ravishments of wine and revelry. If it is melancholy to see an individual drowning his sorrows in wine, what is it to see cowardly nations dissolving their griefs and disappointments in the lethean forgetfulness of intoxication? And this is the case, not only in the Germanic States, but throughout Central and Western Europe—England in part excepted. It is in view of these facts that I consider the evils flowing from intoxicating drinks in Europe much greater than in the United States. The individual excess may not be so apparent and repulsive as in our own country, where there is more activity and more complete exhaustion; where men establish new empires, kill polar bears, and get drunk, with the same national enthusiasm. But Heaven preserve us from sinking into the Dead Sea of European intemperance, involving the loss of both morality and liberty!

Intemperance is astonishingly prevalent among the Sclave tribes on the lower Danube. The Serbs drink their wretched *Slivovitza*, the Wallachs the vinegar-wine, the Greeks and Gipsies dissolve their sorrow in Raki—the vilest possible counterfeit of Brandy. The Wallachs also plant a plum tree at the head, and another at the foot of every grave, from the fruit of which they distill an odious but much-loved liquor: "a very literal illustration," says Paget, "of seeking consolation from the tomb."

The Koran is a judicious code of health applied on a magnificent scale. While we thank Mohammed for abolishing idolatry—wherever the faith of Islam is received—for destroying caste and combining a certain amount of individual freedom with the traditional despotism of the East, we have also to thank that "sanitary commissioner run mad" for restraining millions from the use of intoxicating drinks, in a climate where the temptation to use them is exceedingly great, and the consequences more fatal than elsewhere. Alcohol decimated the English army in the Orient.

The Koran forbids the use of intoxicating drinks. The law of Islam is, however, capable of elastic interpretation, and there are those among the faithful who contend that the interdiction is leveled against the abuse rather than the use of alcoholic beverages. Notwithstanding modern innovations, the great Moslems condemn their use, even in the form of remedies applied internally or externally, believing with the Prophet that "the sin committed in drinking wine is much greater than the advantage reaped from it." Those who have made the pilgrimage to Mecca are most scrupulous on this point. Generally they will neither smoke, nor drink wine —neither buy nor sell it—nor the implements with which it is made, in order to live by such traffic The sumptuary laws of the Koran are exceedingly strict. "Verily," saith the Prophet, "the fires of hell shall roar like the lowings of a camel, in the bellies of such as eat or drink from vessels of gold and silver." Unfortunately, however, there have been many departures from the early purity of the faith of Islam. The island of Scio was conquered by the Grand Vizier, Kipriuli, on account of the excellence of its wines. Even the dervishes—those pious showmen and cunning Jesuits of the East, are not proof against alcohol. Many of the Sultans have been addicted to intemperance, and the last Padisha, Mahmoud, died of *delirium tremens* actually—though of tubercular consumption officially. One of the first acts of Abdul Medjid was to pour into the Bosphorus several thousand bottles of wine surreptitiously introduced within the walls of the seraglio by the Kisler Aga. Many of the soldiers of Omar Pacha drank freely of Schnapps, a vile liquid invented, they declare, by the devil, long after the promulgation of the Koran, and therefore not forbidden. I do not remember, however, having ever seen more than half a dozen Moslems completely overcome by the influence of *French water.*

There is, doubtless, much intemperance in Turkey that escapes the eye of the traveler, but by no means so much as might be inferred from the following, taken from MacFarlane's Kismet: "They (the Turks) have no sense of moderation. They may sometimes abstain, but they can never refrain. When they drink they invariably do it to excess. I never saw a Turkish gentleman sit down to his dinner with any appetite. The danger of dining with them, is getting fuddled before dinner commences. Their stomachs are deranged and vitiated, and the effects of this way of living are visible on the persons of most of them. I certainly did not expect to find that the habit had spread very widely among the common people; yet, with a few exceptions every Turk we met, in Europe or Asia, would drink Raki without any scruples and quite openly; and it was not often that they would refuse to partake of our wine. Delirium tremens was a malady by no means unknown among them."

I once witnessed rather an amusing scene in Varna—[then a pandemonium of war and crime, of filth and wretchedness]—a scene of which only Western civilization can boast, and which was Oriental only in the accident of its locality. A few dissolute women who spoke the English language, but in whose person Venus and Bacchus had united their worst attractions, were engaged in a drunken brawl in the open street, with a number of Scotch and Irish dragoons The French police were attempting to abate the nuisance, but their words were entirely lost on those abandoned courtezans, following in the track of glorious war. A crowd of long-bearded Mussulmans collected to gaze on that singular *apotheosis* of Western civilization in their midst. I shall not soon forget how those turbanned philosophers did shake their heads, and turn away in disgust, saying: "O Allah, the merciful, deliver us from the faults of our friends. By the beard of the Prophet, one Mussulman maiden is worth more than seven of the most beautiful daughters of the unbelievers."

The use of opium is by no means so common among the Turks as I supposed it to be. *Theriakee*, signifying a lover of opium, is a term of reproach. The introduction of alcoholic drinks has doubtless had some influence in diminishing its consumption, but the great majority of the Turks are satisfied with the milder stimulus of their favorite Mocha Nectar.

But now and then in the vicinity of the *Theriakee Tcharthee,* in Stamboul, may be seen a Turk whose pallid brassy countenance, emaciated features, sunken eye-balls, glistening in their sockets, plainly mark him as a smoker of the *Madjoon.* In some instances hideous boils break out upon the face and neck of the sufferer, and in others the limbs are paralyzed. Yet they declare that the mental ravishment produced by their favorite drug is cheaply purchased with these horrible sufferings. Those accustomed to its use frequently consume 100 grs. per day.*—*Ohio Med. Jour. and St. Louis Jour.*

---

* De Quincy used 800 grs. of opium per day, and I attended a female in New York, suffering from a most painful cancer, in which 800 grs. were given daily for a period of three weeks.

